# Long-Term Outcomes of ScoliBrace^®^ Treatment in Juvenile Idiopathic Scoliosis: A Case Series of Four Patients

**DOI:** 10.3390/children13101335

**Published:** 2026-09-30

**Authors:** Rosemary Mirenzi, Juan Du Plessis, Nora-Lee Doueihi, Paul A. Oakley, Joanna S. T. Liu, Jeb McAviney

**Affiliations:** 1 ScoliCare, Sydney, NSW 2217, Australia; 2 Private Practice, Newmarket, ON L3Y 8Y8, Canada

**Keywords:** idiopathic scoliosis, juvenile, brace, ScoliBrace, conservative treatment, bracing, TLSO, Cobb angle

## Abstract

**Highlights:**

**What are the main findings?**

- Reductions in primary thoracic Cobb angles were observed in four patients with juvenile idiopathic scoliosis (JIS) treated with ScoliBrace^®^.
- All curves stayed below pre-treatment values at ≥1-year post-weaning follow-up.

**What is the implication of the main findings?**

- Given the substantial complication burden associated with early surgical intervention in juveniles, effective non-surgical options such as bracing may help delay or avoid the need for surgery in this population.
- ScoliBrace^®^ is a candidate non-surgical option for JIS that warrants evaluation in larger, prospective controlled studies.

**Abstract:**

**Background/Objectives**: There is a lack of both guidelines and evidence supporting the non-surgical treatment of juvenile idiopathic scoliosis (JIS). We present a case series of four juveniles presenting with primary right-thoracic curve types who were treated with the ScoliBrace^®^. **Methods**: Four patients with JIS from a single scoliosis clinic database who met predefined inclusion and exclusion criteria were included in this case series. All patients were braced with the ScoliBrace^®^, a three-dimensional rigid brace designed to target the deformity using Mirror Image^®^ postural over-correction. All patients were initially prescribed full-time (23 h/day) bracing; three patients later transitioned to part-time wear. Cobb angle and angle of trunk rotation (ATR) measures were monitored at 90-day intervals after a one-month in-brace check. **Results**: Patients had a mean (± standard deviation) initial age of 7.2 ± 1.5 years and were followed for a mean of 8.8 ± 1.7 years, including 22.5 ± 15.2 months of post-weaning monitoring. At final follow-up, the primary thoracic curve Cobb angle was improved by a mean of 21.5 ± 12.6°, and ATR by 7.5 ± 2.9°. **Conclusions**: The ScoliBrace^®^ brace and management protocol was associated with an average 21.5° Cobb angle reduction in primary right-thoracic scoliosis curves in four juvenile patients with idiopathic scoliosis. Given the small, uncontrolled sample, these findings should be interpreted as preliminary; they support further evaluation of the ScoliBrace^®^ in larger, prospective controlled studies.

## 1. Introduction

Scoliosis, a three-dimensional spinal deformity characterized by lateral curvature and vertebral rotation, presents significant challenges during juvenile development, particularly in cases of idiopathic scoliosis (IS), which constitutes approximately 80% of pediatric scoliosis diagnoses [1]. Juveniles, defined as individuals aged 4 to 10 years, face an elevated risk of curve progression due to their substantial remaining growth potential, with the pubertal growth spurt being a critical period for progression [2]. This rapid skeletal development can worsen spinal deformities, potentially leading to surgical intervention if the Cobb angle surpasses 40–50 degrees [3]. In fact, JIS curves exceeding 30° at the onset of puberty carry an almost 100% likelihood of progression to surgery [4], emphasizing the need for active treatment and management.

Bracing has emerged as a non-surgical strategy to manage adolescents with idiopathic scoliosis (AIS), with the intention of halting progression, enhancing postural symmetry, and potentially reducing curve magnitude, thus delaying or completely avoiding invasive procedures [3,5]. The ScoliBrace^®^, a custom-designed, 3D-corrected orthosis, has gained prominence for its patient-specific design, which may improve comfort and treatment outcomes [6]. Described as a rigid, three-dimensional, over-corrective thoracolumbosacral orthosis (TLSO) that utilizes a “Mirror Image^®^” approach to guide the spine into an over-corrected posture, the ScoliBrace^®^ leverages advanced 3D scanning and computer-aided design to maximize spinal straightening within the limits of patient flexibility [6,7]. The Mirror Image^®^ concept emphasizes aligning the spine to its symmetrical ideal through targeted corrective forces, a method shown to improve Cobb angles and reduce rotational deformity in scoliosis patients [7,8]. Additionally, a pilot study conducted in Singapore with 30 female adolescents with idiopathic scoliosis, utilizing the ScoliBrace^®^, demonstrated significant curve reduction and maintenance below surgical thresholds, supporting its potential as a non-surgical intervention [5].

Despite its clinical significance and clear link to severe, long-term consequences, research on JIS is scarce, with a notable lack of studies, clear guidelines, and evidence-based protocols for non-operative treatments including bracing [9]. Although bracing is well established for AIS [3], evidence in JIS remains limited and cannot be directly extrapolated from AIS studies. A 2025 systematic review and meta-analysis of 16 studies on factors contributing to JIS bracing success found that a pre-brace Cobb angle below 30° and full-time brace-wear compliance of at least 18–20 h per day were both significantly associated with successful outcomes, while concluding that the existing JIS literature remains heterogeneous and of generally low quality [9]. Similarly, a systematic review evaluating bracing outcomes in idiopathic early-onset scoliosis to skeletal maturity found only 15 studies meeting eligibility criteria, all of which were observational and carried a high risk of bias, reflecting the limited availability of long-term, JIS-specific bracing data [10]. This gap is clinically significant, as young children who progress despite conservative management may ultimately require surgical intervention, which in this age group carries a substantial complication burden and often necessitates repeated procedures to accommodate ongoing growth [11], highlighting the clinical importance of effective non-surgical treatment options.

JIS patients are younger than AIS, have greater remaining growth, and demonstrate higher rates of curve progression [12], which may alter both the natural history and the responsiveness to conservative interventions [13]. This knowledge gap reveals the critical importance of exploring effective interventions for JIS to better inform clinical practice and improve outcomes for this pediatric population. This case series adds to the limited body of JIS bracing literature by evaluating ScoliBrace^®^, a custom, over-corrective TLSO, in a JIS population—an under-represented curve/age group in existing brace-design studies—with outcomes reported through post-weaning follow-up, including three of four patients followed to skeletal maturity. This addresses a specific gap in the literature, as such long-term follow-up is infrequently reported in JIS bracing studies [10]. The aim of this study is thus to present a retrospective case series of four patients with JIS managed using the ScoliBrace^®^ protocol, describing changes in curve magnitude and postural symmetry, and reporting the sustainability of these outcomes at least 1 year post-weaning.

## 2. Methods

This case series represents a prospectively collected, retrospective report on patients who presented with JIS to a spinal rehabilitation facility. Between 1 November 2024 and 5 December 2024, a single-center prospective database of 271 patients with scoliosis was retrospectively reviewed to identify all individuals with JIS who met predefined inclusion and exclusion criteria. One patient (Patient 2), who had not yet met the ≥12-month post-treatment follow-up criterion at the time of review, was identified as potentially eligible and was included once this criterion was subsequently met through routine clinical follow-up; data collection for this patient continued through mid-2025. The full participant selection process is summarized in Figure A1. All eligible patients (*n* = 4) were included in this case series. All patients and their parents consented to publication, and ethics approval for this research was granted by Macquarie University (reference: 5201826252998, 8 August 2018).

### 2.1. Inclusion/Exclusion Criteria

Patients were included if they met all of the following inclusion criteria: (1) Idiopathic scoliosis diagnosis between the ages of 4–10 years indicative of JIS (International Classification of Diseases, Tenth Revision (ICD-10) M41.1); (2) posteroanterior (PA) radiographic exam revealing a primary right-thoracic curve pattern with secondary or tertiary lumbar and/or upper thoracic curves; (3) Cobb angle 10 degrees or more on PA X-ray; (4) angle of Trunk Rotation (ATR) recorded at all examinations; and (5) files containing follow-up exams ≥ 12 months post-treatment to assess post-weaning stability. Files were excluded if the patient/parent/carer refused informed consent; there was an underlying neuromuscular, congenital or additional factor complicating an “idiopathic” diagnosis; or if the patient was simultaneously participating in an external treatment program.

### 2.2. Outcome Measures

#### 2.2.1. Radiographic Analysis

PA thoracolumbar spinal radiographs were captured at pre-treatment, ScoliBrace^®^ fitting (in-brace), and at post-treatment assessments. The Cobb method of measurement was used to examine all PA radiographs [14], where the superior endplate of the uppermost vertebra and the inferior endplate of the lowermost vertebra were digitized; the intersection of the lines at right angles to the lines created from the two endplate lines is the Cobb angle. Inter-observer reliability of Cobb angle measurements was assessed among three scoliosis clinicians using a two-way random-effects intraclass correlation coefficient for single measurements (ICC(2,1)), across all 20 radiographs (five per patient, from pre-treatment through final follow-up). Assessors were blinded to patient identity, treatment stage, and prior measurements, and performed their assessments independently. A reduction in Cobb angle of greater than 5° is defined as the threshold considered to be a valid and clinically significant improvement in scoliotic curve [15,16,17].

#### 2.2.2. Standing Posture Assessment

Photographic posture assessments were performed prior to treatment, at the ScoliBrace^®^ fitting (in-brace), as well as at post-treatment exams. The patient was positioned in a neutral, standing, weight-bearing posture against a wall grid, allowing for easy visual identification of postural misalignments (i.e., rotations and translations of the head, thorax, and pelvis from a normal position in a three-dimensional coordinate system).

#### 2.2.3. Adam’s Forward Bend Test and Angle of Trunk Rotation

The Adam’s Forward Bend Test was performed as part of routine clinical examination. During this test, the ATR is assessed using a scoliometer that is moved along the patient’s spine, and the maximum angle is measured at the scoliotic curve of interest. This examination helps identify the maximum trunk rotation in different spinal regions offering insight into the three-dimensional deformity [18], with 0° as the ideal value. Coelho et al. (2013) reported a strong correlation between scoliometer readings and radiographic measurements (r = 0.7 with *p* < 0.05) [19]. ATR measurement at the main thoracic and thoracolumbar regions appears to be the most informative for identifying children who may require further scoliosis assessment [20]. In their study of patients with idiopathic scoliosis, Coelho et al. (2013) also reported that an ATR referral threshold of 5° had 87% sensitivity for detecting curves >10° and 100% sensitivity for detecting curves >20° [19]. Bunnell proposed a minimum ATR threshold of 7° for referral, reducing false positives relative to a 5° threshold [21]. Stable ATR measurements have been demonstrated to offer a safe, non-invasive, and cost-effective alternative to serial radiographs for the clinical monitoring of AIS [18]. It is not intended for diagnostic use, but rather it provides an indication of the need for an X-ray [22]. In some studies, a change of at least 2° in ATR between consecutive assessments has been considered indicative of progression or improvement [23], whereas others have considered a change of at least 3° to be significant [18].

### 2.3. ScoliBrace^®^ TLSO Bracing

Patients were treated with a custom-molded, rigid TLSO (ScoliBrace^®^; Canfit, version 17). Each orthosis was individually designed using three-dimensional scanning and computer-aided design and manufacturing (CAD/CAM) [24,25]. The ScoliBrace^®^ incorporates a “Mirror Image^®^” approach [6,24,26,27], an over-corrective approach that positions the patient opposite to their predominant scoliosis-induced posture. This over-corrected positioning uses axial elongation and counter-stress forces to facilitate three-dimensional spinal and postural correction through the principle of spinal coupling, aiming to achieve straightening within the limits of the patient’s spinal flexibility [28].

Patients were initially prescribed full-time ScoliBrace^®^ wear for 23 h per day. Brace wear was subsequently reduced to part-time use according to the treating clinician’s protocol and clinical progress. A new brace was prescribed when growth and changes in curve magnitude or alignment resulted in the existing brace no longer providing an appropriate fit or corrective position. Active treatment duration was defined as the interval from brace initiation to the end-of-bracing assessment. Total follow-up duration was defined as the interval from the initial consultation to the final follow-up assessment. For clarity, “end-of-bracing” refers to the assessment conducted at the completion of the bracing period, during which the health measures collected at baseline were repeated. “Final follow-up” refers to the later assessment conducted after the patient had an extended period out of the brace, marking the end of the monitoring period. Continuous variables are presented as mean ± standard deviation (SD), with ranges reported where appropriate.

#### 2.3.1. Bracing Protocol

No juvenile-specific consensus guidelines for bracing decision-making currently exist. Bracing protocols in this study were adapted from general idiopathic scoliosis guidelines, primarily the SOSORT 2016 guidelines for orthopedic and rehabilitation treatment of idiopathic scoliosis during growth [29], which include recommendations for juvenile idiopathic scoliosis but are supported predominantly by evidence from adolescent idiopathic scoliosis. In accordance with the treating clinic’s established protocol, the initial in-brace radiograph was used to assess the extent of Cobb angle correction, with a target of approximately 30–50% reduction from the out-of-brace measurement. Full-spine radiographs were reviewed to assess the contour of the ribs alongside the degree of in-brace correction. Subsequent brace modifications were guided by the observed corrections, radiographic morphology, postural changes, patient comfort, and physical growth.

Comfort and any adverse effects were assessed at every follow-up session following brace fitting, with clinicians trained to routinely ask about comfort and make modifications as needed at each visit. Follow-up assessments occurred at 1 month post-fitting, 3 months post-fitting, and at 3-monthly intervals thereafter. In-brace radiographs were obtained at the first follow-up visit and repeated only if major brace modifications were subsequently required. Out-of-brace radiographs were obtained at the second follow-up visit and at 6-monthly intervals thereafter, following a standardized period of brace removal prior to imaging.

#### 2.3.2. Brace Weaning

Weaning from full-time bracing was considered once patients met the following criteria: Risser stage 4 or greater, no increase in height over the preceding 6 months, and, for female patients, at least 2 years post-menarche. Weaning was undertaken gradually over several months, with brace-wear hours progressively reduced, followed by a structured period of brace removal prior to a final out-of-brace radiograph to confirm curve stability before bracing was formally discontinued. For Patients 2–4, weaning commenced after full skeletal maturity (Risser Stage 5, Sanders Stage 8) [30,31] had been reached and the curve had remained stable, without progression for 6 months. Brace wear was then gradually reduced and then removed. A brace holiday (cessation of bracing) was used for Patient 1 when substantial curve correction (to under 10°) was achieved, despite not meeting the standard weaning criteria of Risser stage 4 or greater, as she remained Risser 0.

## 3. Results

Re-evaluations occurred 1 month following the brace fitting session for an in-brace X-ray and fit check, and then every 90 days thereafter. Active treatment duration was 6.9 ± 2.8 years (range 2.9–9.1), and patients were monitored for a total of 8.8 ± 1.7 years (range 6.6–10.5). Post-weaning monitoring duration was thus 1.9 ± 1.3 years (range 1.0–3.8). Inter-observer reliability of Cobb angle measurements was high (ICC(2,1) = 0.96), 95% CI [0.936–0.977], (*p* < 0.001) across all curve measurements in the 20 radiographs.

The ScoliBrace^®^ brace and management protocol was well tolerated by all patients. It should be noted that some degree of constriction is an inherent part of the brace’s corrective mechanism, and patients may be expected to experience an initial period of adjustment. Minor, transient discomfort was reported in all four patients, some of which was attributable to growth over the treatment period, and was managed with brace modifications such as panel adjustment, added padding, trimline contouring, or shoulder paddle trimming. Beyond routine adjustments to accommodate growth, no major modifications were required in any patient.

### 3.1. Patient 1

On 30 April 2018, a 5-year-old female (Figure 1A) presented with JIS, having originally been diagnosed in November 2017 at approximately 4.7 years of age. She had a right-thoracic major curve (Cobb angle T5 to T11—apex at T8) measuring 30° with a secondary left lumbar curve (Cobb angle T12-L4 (L2/3)) measuring 22° (Figure 1B). The patient had a pre-treatment right-thoracic ATR measuring 10°, and a Risser score of 0 (Table 1). Patient 1 initially commenced a full-time ScoliBrace^®^ program prescribed for 23 h per day. At the initial ScoliBrace^®^ fitting (1 month after the initial consultation, Figure 1C,D), the in-brace radiograph showed correction of the major curve (T5–T11 (T8)) measuring 11° (63.3% reduction, Figure 1D) and secondary curve (T12-L4 (L2/3)) reduced to 3° (86.4% reduction, Figure 1D). After the major curve remained stable at 11° for 6 months, brace wear was progressively reduced to part-time and then night-time use. The overall bracing period lasted approximately 34 months, concluding on 8 March 2021. Patient 1 was fitted with a new ScoliBrace^®^ TLSO in June 2019 and October 2020 (Figure 1E) due to skeletal growth and curvature improvement. The final in-brace measurements showed improved major and secondary curves to 2° and 0° (93.3% and 100% reduction) respectively (Figure 1F).

After brace wear had been reduced to night-time use, the curve stabilized at approximately 10°, allowing a brace holiday to commence. Bracing treatment was concluded on 8 March 2021 and re-assessment showed improved coronal balance and ATR on posture (Figure 1G). At the conclusion of bracing treatment, the out-of-brace radiograph showed an improvement in the major and minor curve Cobb angles to 12° and 11° (60% and 50% reduction) respectively, (Figure 1H). The ATR in the primary thoracic region improved to 6°, which was maintained from ~1-month follow-up (40% reduction) indicating correction in trunk symmetry. Following the conclusion of bracing, the patient was monitored for approximately 4 years and participated in ScoliBalance^®^-based Physiotherapeutic Scoliosis-Specific Exercise (PSSE) to help maintain the correction, as described by Marchese et al. (2024) [6]; as such, her post-weaning outcome cannot be attributed to bracing alone.

During this period, the established 15° Cobb angle threshold for recommencing brace treatment was not reached. Re-assessment on 9 December 2024, 45 months post-brace treatment and representing the final follow-up for this patient, showed posture and curve improvement and stabilization of both primary and secondary curves measuring 6° and 7° (80% and 68.1% reduction) respectively (Figure 1I,J). The 45-month post-treatment ATR in the primary thoracic region remained at 6° (40% reduction from initial ATR). At this final follow-up (11.85 years of age), the patient had not yet reached full skeletal maturity (Risser 0); the sustained correction observed at this stage is therefore a positive result, but should be considered interim rather than a definitive long-term outcome. Additionally, as PSSE was introduced after the conclusion of bracing, the post-weaning outcome in this patient cannot be attributed solely to the effect of brace treatment. Cobb angle and ATR changes across treatment timepoints for Patient 1 are also presented graphically in Figure A2 (Appendix A.2).

### 3.2. Patient 2

On 19 January 2017, a 7-year-old female (Figure 2A) presented with JIS, having originally been diagnosed in March 2016, at approximately 5.8 years of age. She had a primary right-thoracic curve (T6 to T12 (T9)) measuring 37°, a secondary left upper thoracic curve (T1–T5 (T3)) measuring 27° and tertiary left lumbar hemicurve (Cobb angle L1–L5 (L3)) measuring 20° (Figure 2B). The patient had a pre-treatment right-thoracic ATR measuring 17°, and a Risser score of 0 (Table 1). Patient 2 was treated for 88 months on a full-time ScoliBrace^®^ program (23 h/day). At the initial ScoliBrace^®^ fitting (1 month after the initial consultation, Figure 2C,D), the in-brace radiograph showed correction of the primary, secondary and tertiary curves measuring 8°, 21° and 9° (78.4%, 22.2%, and 55% reduction from the initial Cobb angle), respectively. Patient 2 was fitted with a new ScoliBrace^®^ TLSO in July 2018, February 2021 and July 2022 (Figure 2E) due to skeletal growth and curvature improvement. The final in-brace measurements showed Cobb angles of 14°, 23° and 0° for the primary, secondary and tertiary curves, (62.2%, 14.8%, and 100% reduction) respectively (Figure 2F). The lumbar curve was considered a hemicurve; as there was no sloping of both the upper and lower endplates, it was not classified as a true scoliotic curve. Cobb angle and ATR changes across treatment timepoints for Patient 2 are also presented graphically in Figure A3 (Appendix A.2).

Bracing treatment was concluded on 25 June 2024 and the out-of-brace radiographs showed improvement of Cobb angle of the primary, secondary and tertiary curves of 19°, 24° and 0° (a transient in-brace compensatory curve, with no long-standing structural component), 48.7%, 11.1% and 100% reduction, respectively (Figure 2H). The ATR in the primary thoracic region improved to 5° (70.6% reduction), demonstrating improvement in trunk symmetry. The patient continued to be monitored post-brace treatment and re-assessment was conducted on 8 July 2025, approximately 12 months post-brace treatment, and showed posture and curve stabilization with the primary, secondary and tertiary curves measuring 22°, 21° and 0° respectively, (again representing a transient in-brace compensatory curve rather than a true scoliotic curve; 40.5%, 22.2% and 100% reduction from initial Cobb angle) (Figure 2I,J). The ~12-month post-treatment ATR in the primary thoracic region increased to 9° (47.1% reduction from the initial ATR); however, the posture remained stable.

### 3.3. Patient 3

On 13 June 2014, an 8-year-old male (Figure 3A) presented with JIS, having first been diagnosed in November 2013. He had a primary right-thoracic curve (T6–T12 (T10)) measuring 31°, a secondary upper left thoracic curve (T1–T5 (T4)) measuring 27° and a tertiary left lumbar hemicurve (L1–L5 (L3/4)) measuring 20° (Figure 3B). The patient had a pre-treatment right-thoracic ATR measuring 15° and a Risser score of 0 (Table 1). Patient 3 was treated for 83 months on a full-time ScoliBrace^®^ program (23 h/day) and 25 months on a part-time ScoliBrace^®^ program (10–18 h per day). At the initial ScoliBrace^®^ fitting (1 month following the initial consultation, Figure 3C), the in-brace radiograph showed correction of the primary, secondary and tertiary curves to 17°, 16° and 12° (45.2%, 40.7%, and 40% reduction), respectively (Figure 3D). Patient 3 was fitted with a new ScoliBrace^®^ TLSO on June 2015, July 2016, September 2018, December 2019 and April 2021 (Figure 3E) due to skeletal growth and curvature improvements. The final in-brace measurements showed improvements in the primary, secondary and tertiary curves to 17°, 20° and 0° (45.2%, 25.9%, and 100% reduction), respectively (Figure 3F).

Bracing treatment was concluded on 12 July 2023 and re-assessment showed improved posture (Figure 3G). The three scoliotic curves, primary, secondary and tertiary, measured 20°, 22° and 11° (35.5%, 18.5% and 45% reduction), respectively (Figure 3H). The ATR in the primary thoracic region improved to 10° (33.3% reduction). Re-assessment on 11 December 2024, 17 months post-brace treatment, showed posture and curve stabilization with the primary, secondary and tertiary curves measuring 22°, 22° and 16°, respectively (29%, 18.5% and 20% reduction from initial Cobb angle, respectively, Figure 3I,J). The 17-month post-treatment ATR in the primary thoracic region reduced to 8° (46.7% reduction from initial ATR). Cobb angle and ATR changes across treatment timepoints for Patient 3 are also presented graphically in Figure A4 (Appendix A.2).

### 3.4. Patient 4

On 11 August 2013, an 8-year-old female (Figure 4A) presented with JIS, having first been diagnosed at approximately 6 years of age. She had a primary right-thoracic curve (T6–T11 (T9)) measuring 42° and a secondary left lumbar curve (T12-L4 (L2)) measuring 30° (Figure 4B). The patient had a pre-treatment right-thoracic ATR measuring 17° and a Risser score of 0 (Table 1). Patient 4 was treated for 36 months on a full-time ScoliBrace program (23 h/day) and 54 months on a part-time ScoliBrace program (12–18 h per day). At the initial ScoliBrace^®^ fitting (1 month after the initial consultation, Figure 4C), the in-brace radiograph showed correction of the primary curve to 19° (54.8% reduction) (Figure 4D), and the lumbar curve measured 20° (33.3% reduction). Patient 4 was fitted with a new ScoliBrace^®^ TLSO on December 2014, June 2017 and March 2019 (Figure 4E) due to skeletal growth and curvature improvement. The final in-brace measurements show an improvement to 7° and 9° for the primary and secondary curves, (83.3% and 70% reduction) respectively (Figure 4F).

Brace treatment was concluded on 5 October 2021 and re-assessment showed improved posture (Figure 4G). At the conclusion of bracing treatment, out-of-brace radiographs showed improvement of the primary and secondary curves of 7° and 10° (83.3% and 66.7% reduction) respectively (Figure 4H). The ATR in the primary thoracic region improved to 7° (58.8% reduction). A follow-up assessment on 17 January 2023, 15 months post-brace treatment, showed postural symmetry (Figure 4I) and the primary and secondary curves demonstrated stabilization measuring 4° and 11° (90.5% and 63.3% reduction from the initial Cobb angle) respectively (Figure 4J). The ATR in the primary thoracic region reduced to 6° (64.7% reduction from the initial ATR). Cobb angle and ATR changes across treatment timepoints for Patient 4 are also presented graphically in Figure A5 (Appendix A.2).

## 4. Discussion

This report documents the outcomes of four juvenile patients who were treated for scoliosis with the ScoliBrace^®^ bracing protocol. All patients demonstrated a change exceeding the 5° radiographic threshold in the primary thoracic curve [15,16,17]; in fact, the average reduction was 21.5° ± 12.6° (range 9–38°). Two patients showed a reduction in the lumbar curve, with an average reduction of 17 ± 2.8° (range 15–19°). Two patients also had a mean reduction of 5.5 ± 0.7° in their secondary upper thoracic curves (range 5–6°). Clinically, reductions of this magnitude are relevant because larger curves are associated with a greater risk of progression to surgical thresholds. Similarly, the mean ATR improvement of 7.5 ± 2.9° (range 4–11°) exceeds the 2–3° threshold for meaningful change [18], indicating relevant improvement in trunk symmetry. Both thresholds exceed the reported measurement error for Cobb angle (3–5°) and scoliometer ATR (2–3°), indicating the changes are unlikely to reflect measurement variability alone. This is particularly relevant in juvenile patients, who face a longer period of remaining growth and a higher risk of progression to surgical thresholds than adolescent-onset cases.

The ScoliBrace^®^ brace and management protocol was well tolerated across all patients. Comfort and any adverse effects were assessed at every follow-up session following brace fitting. Minor, transient discomfort was reported in all patients (mild chest, neck, or trim-related discomfort), each resolved with brace modifications such as panel adjustment, added padding, or trimline contouring. Beyond routine adjustments to accommodate growth, no major modifications were required in any patient. The observed reductions in Cobb angle and ATR occurred during a bracing-only protocol, with the exception of PSSE introduction after brace cessation for Patient 1. This is consistent with the in-treatment changes reflecting the effect of the ScoliBrace^®^ regimen; however, without a comparator group, this uncontrolled case series cannot definitively establish that attribution.

Although preliminary, the results from this case series of patients with JIS align with earlier investigations into the prescription of the ScoliBrace^®^ for adolescents with idiopathic scoliosis, specifically, with regard to reducing the Cobb angle [5,32]. These results also align with recent studies showing the effectiveness of part-time and night-time bracing for patients with JIS [33,34]. Rymond et al. (2026) found that part-time bracing prevented curve progression and produced measurable improvement against an observational comparison cohort [33], while Simon et al. (2026) followed a JIS cohort to skeletal maturity and found that night-time detorsion bracing helped 49% of patients avoid surgery [34]. The larger-magnitude corrections achieved here across all four patients (mean 21.5° vs. 3.2° improvement reported by Rymond et al. (2026) [33]) may reflect the full-time (23 h/day) wear prescribed here, as compared to the part-time regimen. A dose–response relationship between brace-wearing time and curve correction is well established in AIS [35,36]; although this has not been directly confirmed in JIS, a comparable effect is plausible given the shared biomechanical principles underlying bracing across both populations.

This series included patients with JIS who had a primary right-thoracic curve, and all cases presented with an initial curve of at least 30°. These characteristics represent a less favorable prognostic profile for bracing, as a thoracic curve pattern and initial Cobb angle (≥30°) have been associated with reduced bracing success and increased risk of curve progression [9]. Notably, this case series did not include a control group, and the true unbraced trajectory of these patients’ curves cannot be known. However, the literature on progression offers a reasonable indication of the risk associated with curves of this magnitude. In a cohort of 205 patients with juvenile idiopathic scoliosis followed to skeletal maturity, curves exceeding 30° at the onset of puberty carried a 100% likelihood of progressing to spinal fusion, compared with 75% for curves of 21–30° and 15.6% for curves of 20° or less [4]. As all patients in the present series presented with curves of 30° or greater, they would be expected to fall into this highest-risk category. The results reported here thus diverge from these prognostic expectations: despite all four patients meeting both criteria, favorable long-term correction was achieved, with Patient 1’s outcome representing an interim result given that skeletal maturity had not been fully reached at the final follow-up. These results suggest that unfavorable curve characteristics at presentation may not preclude a positive outcome with high-compliance, full-time bracing.

Another important factor in determining the long-term outcomes for brace prescription for JIS patients involves the compliance to brace wearing [9,13]. Given the high likelihood of curve progression and need for spinal surgery in patients with progressive thoracic JIS patterns [37], any brace-wearing regimen aiming to avoid surgical intervention must involve a team approach that includes child and parent buy-in [38]. All patients were initially prescribed full-time (23 h/day) wear, though adherence was self-reported and not objectively measured, which may introduce selection bias. Greater brace-wear duration is associated with more favorable Cobb angle outcomes in the prior literature, suggesting a dose–response relationship between brace wear and curve improvement [3,5]. Whether the prescribed wear time of 23 h per day may have contributed to the outcomes observed here cannot be confirmed without adherence data. This limits generalizability to patients with lower adherence, as the relationship between compliance and outcome could not be directly assessed. Future research with the ScoliBrace^®^ should incorporate objective compliance monitoring (e.g., temperature sensors) across larger populations to examine the relationship between wear time and patient outcomes.

The preliminary evidence from this series extends the application of this ScoliBrace^®^ technology beyond its initial design for AIS [6,24,32] to JIS. Retention of curve reduction below pre-treatment values at the 1-year follow-up was noteworthy given the highly progressive nature of JIS and the substantial risk of progression to surgical magnitude during growth [4,37], although some correction was lost after weaning in two patients. In Patient 1, the introduction of ScoliBalance^®^ PSSE following brace weaning constitutes a confounding variable in interpreting the durability of post-treatment correction at the final follow-up, as PSSE was not introduced in Patients 2–4. Further, Patient 1 remained skeletally immature at the final follow-up (Risser 0, at 12 years of age) and had substantial growth remaining, unlike Patients 2–4, who had reached skeletal maturity by the final follow-up; her post-weaning stability should therefore be weighted more cautiously than that of the other patients.

The observed radiographic improvements may reflect the efficacy of the ScoliBrace^®^’s customized design, which is tailored to the patient’s individual curve pattern and intended to promote three-dimensional spinal and postural realignment. The brace is a rigid, custom-molded TLSO based on the Mirror Image^®^ approach, which aims to position the patient in an over-corrected posture opposite to the direction of the deformity [6,24,26,32]. This approach is intended to maximize in-brace correction by using three-dimensional spinal coupling and the patient’s available spinal flexibility and overall mobility, rather than relying solely on conventional three-point pressure [6,8]. Although the mechanisms underlying curve progression are not fully established, treatment should acknowledge that persistent asymmetric spinal loading may contribute to differential vertebral growth and progression of deformity [39]. If curve progression is partly driven by asymmetric spinal loading, the associated shear forces may play an important role. Accordingly, even partial correction with bracing could reduce the asymmetric loading and shear forces acting on the spine, potentially decreasing the likelihood of further curve progression [40]. The degree of early in-brace correction may therefore be clinically relevant, as a greater initial correction may reflect a more favorable long-term prognosis, particularly among patients with good brace compliance [41]. It is noteworthy that all JIS patients achieved a significant in-brace reduction at the one-month assessment (45–78%) and this translated to durable curve reduction at follow-up.

Future research into the non-surgical management of JIS using the ScoliBrace^®^ is warranted. It is suggested that a trial be conducted where various factors are accounted for, including brace-wear compliance, inclusion of PSSE, etc. Since challenges to brace treatment for this age go beyond physical factors [30,42]; mental well-being and other social factors were not assessed but are suggested to be included in future research. This study’s limitations stem from its retrospective design and small sample of four patients. As a case series without a comparator or untreated control group, attribution of the observed improvements to bracing alone cannot be established, and the design is susceptible to selection bias, given that only patients with ≥12 months of post-weaning follow-up were eligible for inclusion. Further, other scoliosis curve types should be assessed. The curve pattern studied—a primary right-thoracic curve—reflects the most common presentation in JIS, but findings may not generalize to other curve types. The sample was also heterogenous in secondary and tertiary curve involvement, and treatment and follow-up duration, limiting the ability to draw uniform conclusions across patients. Finally, although patients were initially prescribed full-time wear, adherence was self-reported and not objectively verified, so actual wear time may have been overestimated. If true compliance was lower than reported, the corrections observed here may understate what could be achieved with verified full-time adherence.

## 5. Conclusions

In this retrospective case series of four patients with JIS, the ScoliBrace^®^ bracing and management protocol was associated with an average 21.5° reduction in primary right-thoracic Cobb angles. This case series provides preliminary evidence for the ScoliBrace^®^ as a non-invasive intervention for JIS. However, given the small sample size and the likelihood that high compliance positively influenced outcomes, larger, prospective controlled studies are needed to confirm the effectiveness and generalizability of ScoliBrace^®^ as a non-surgical treatment option for this population.

## Figures and Tables

**Figure 1:**
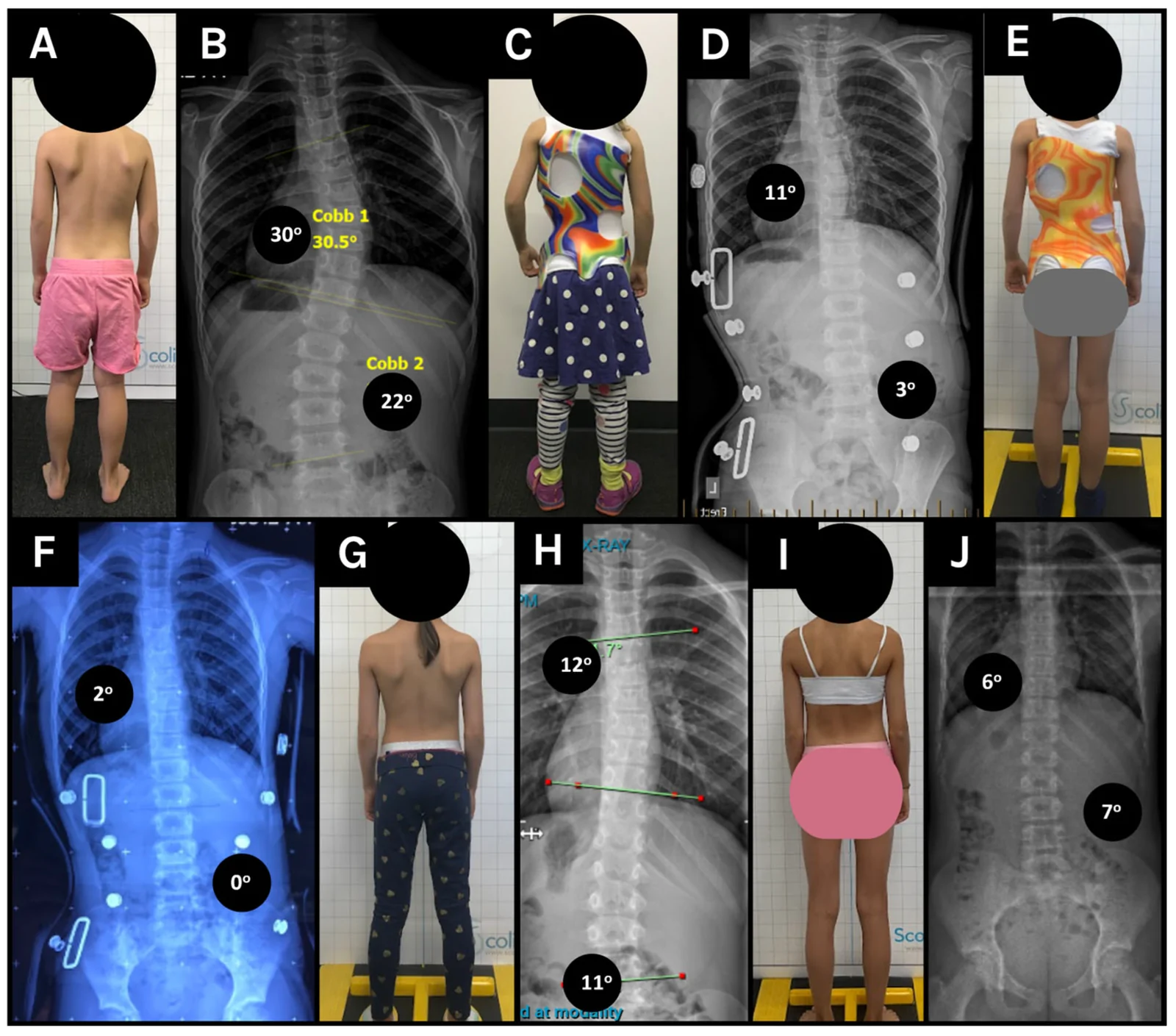
Patient 1. (**A**) Pre-treatment posteroanterior (PA) posture photograph; (**B**) pre-treatment PA radiograph; (**C**) PA initial in-brace posture photograph; (**D**) PA initial in-brace radiograph; (**E**) PA final in-brace posture photograph; (**F**) PA final in-brace radiograph; (**G**) end-of-bracing PA posture photograph; (**H**) end-of-bracing PA radiograph; (**I**) 45-month post-treatment PA posture photograph; and (**J**) 45-month post-treatment PA radiograph.

**Figure 2:**
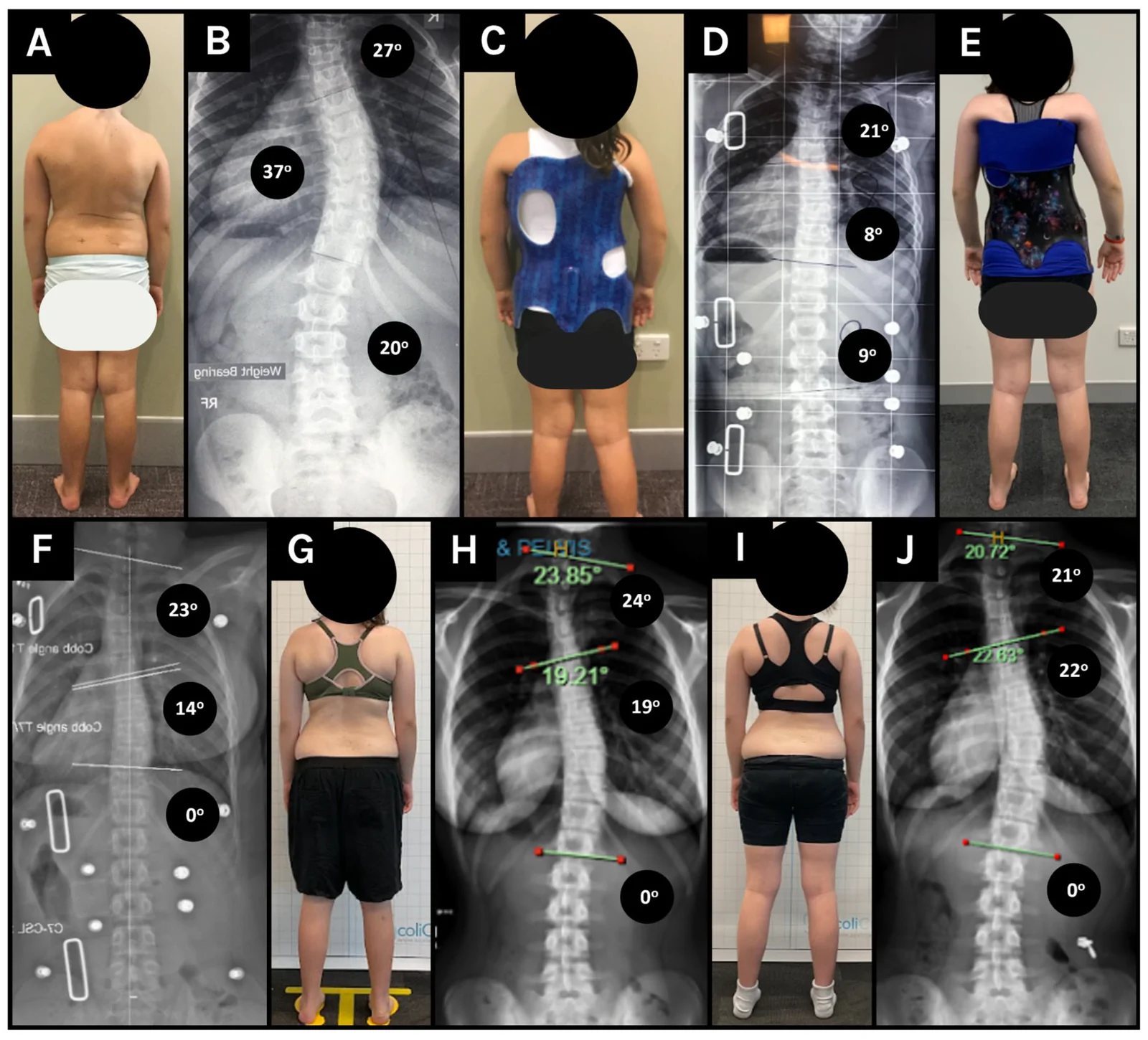
Patient 2. (**A**) Pre-treatment PA posture photograph; (**B**) pre-treatment PA radiograph; (**C**) PA initial in-brace posture photograph; (**D**) PA initial in-brace radiograph; (**E**) PA final in-brace posture photograph; (**F**) PA final in-brace radiograph; (**G**) end-of-bracing PA posture photograph; (**H**) end-of-bracing PA radiograph; (**I**) 12-month post-treatment PA posture photograph; and (**J**) 12-month post-treatment PA radiograph.

**Figure 3:**
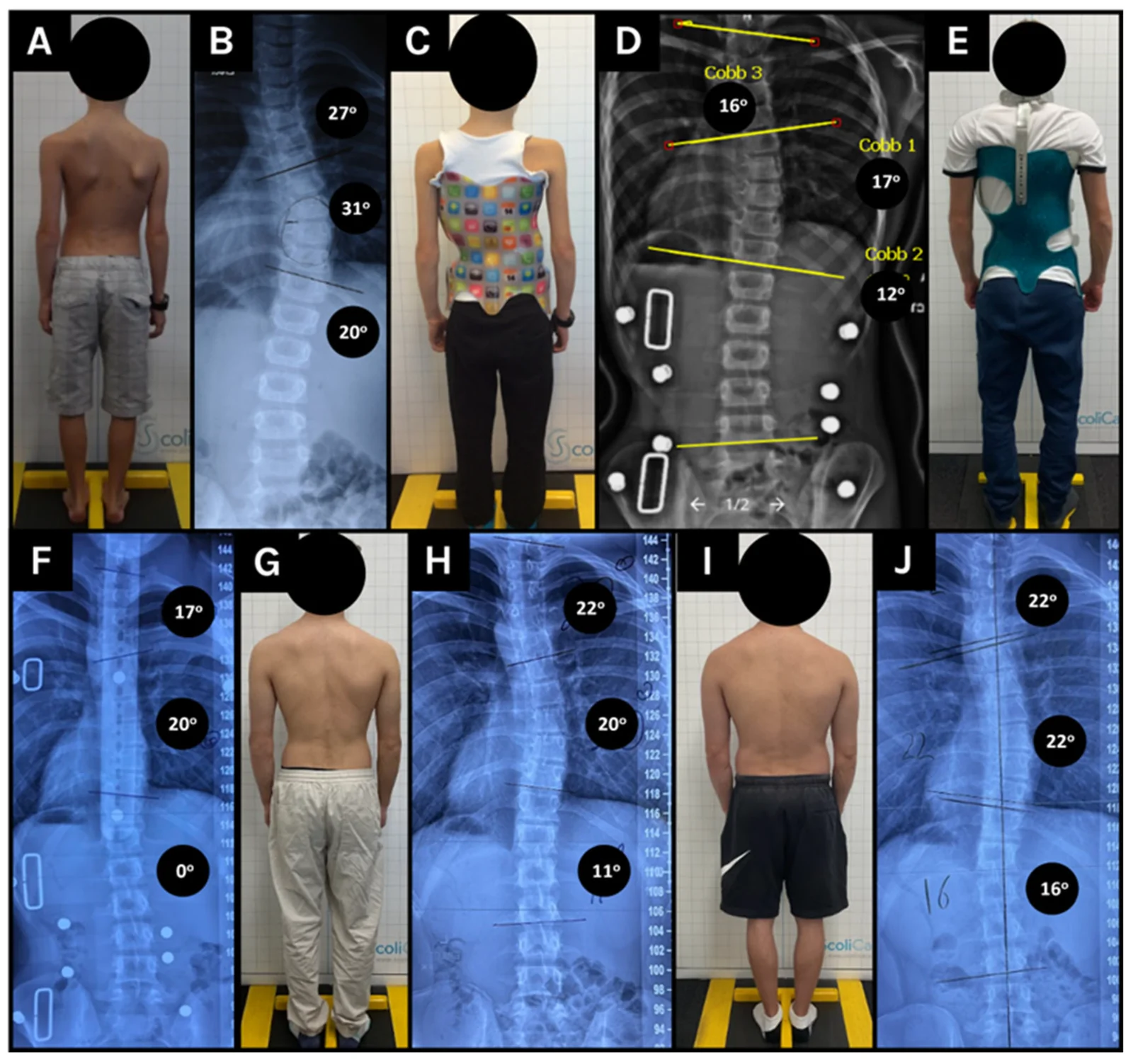
Patient 3. (**A**) Pre-treatment PA posture photograph; (**B**) pre-treatment PA radiograph; (**C**) PA initial in-brace posture photograph; (**D**) PA initial in-brace radiograph; (**E**) PA final in-brace posture photograph; (**F**) PA final in-brace radiograph; (**G**) end-of-bracing PA posture photograph; (**H**) end-of-bracing PA radiograph; (**I**) 17-month post-treatment PA posture photograph; and (**J**) 17-month post-treatment PA radiograph.

**Figure 4:**
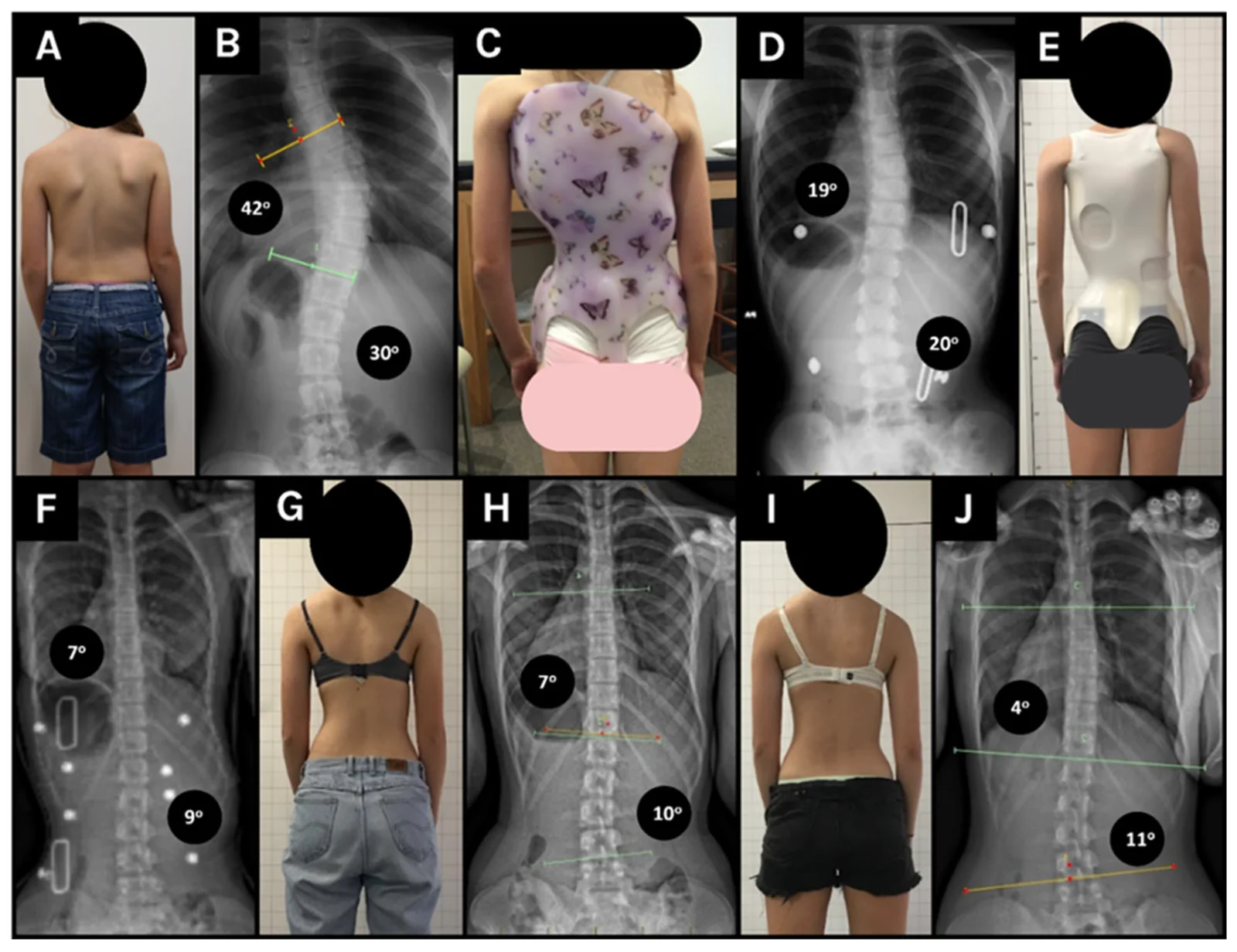
Patient 4. (**A**) Pre-treatment PA posture photograph; (**B**) pre-treatment PA radiograph; (**C**) PA initial in-brace posture photograph; (**D**) PA initial in-brace radiograph; (**E**) PA final in-brace posture photograph; (**F**) PA final in-brace radiograph; (**G**) end-of-bracing PA posture photograph; (**H**) end-of-bracing PA radiograph; (**I**) 15-month post-treatment PA posture photograph; and (**J**) 15-month post-treatment PA radiograph.

**Table 1:** Participant sex as well as age, Cobb angles and Angle of Trunk Rotation (ATR) at key treatment stages.

Outcome Measures	Patient 1	Patient 2	Patient 3	Patient 4	Mean ± SD
Age at Diagnosis Age at Initial Consult End-of-Bracing Age Final Follow-Up Age	4.7 5.2 8.1 11.9	5.8 6.7 14.1 15.2	8.1 8.7 17.8 19.2	6 8.1 16.2 17.5	6.2 ± 1.4 7.2 ± 1.5 14.1 ± 4.3 15.9 ± 3.2
Initial Risser Score Final Follow-Up Risser Score Final Follow-Up Sanders Score	00-	05 8	05 8	05 8	- - -
Sex	F	F	M	F	-
**Primary Right-Thoracic Curve** Pre-Treatment Cobb Angle (°) End-of-Bracing Cobb Angle (°)	30 12	37 19	31 20	42 7	35 ± 5.6 14.5 ± 6.1
Final Follow-Up Cobb Angle (°)	6	22	22	4	13.5 ± 9.8
Δ Cobb Angle (°) (% change)	−24 (80)	−15 (40.5)	−9 (29)	−38 (90.5)	−21.5 ± 12.6
**Lumbar Curve** Pre-Treatment Cobb Angle (°) End-of-Bracing Cobb Angle (°)	22 11	- -	- -	30 10	26 ± 5.7 10.5 ± 0.7
Final Follow-Up Cobb Angle (°)	7	-	-	11	9 ± 2.8
Δ Cobb Angle (°) (% change)	−15 (68.1)	-	-	−19 (63.3)	−17 ± 2.8
**Upper Thoracic Curve** Pre-Treatment Cobb Angle (°) End-of-Bracing Cobb Angle (°)	- -	27 24	27 22	- -	27 ± 0.0 23 ± 1.4
Final Follow-Up Cobb Angle (°)	-	21	22	-	21.5 ± 0.7
Δ Cobb Angle (°) (% change)	-	−6 (22.2)	−5 (18.5)	-	−5.5 ± 0.7
**Lumbar Hemicurve** Pre-Treatment Cobb Angle (°) End-of-Bracing Cobb Angle (°) Final Follow-Up Cobb Angle (°) Δ Cobb Angle (°) (% change)	- - - -	2000−20 (100)	20 11 16 −4 (20)	- - - -	20 ± 0 5.5 ± 7.8 8 ± 11.3 −12 ± 11.3
Pre-Treatment ATR (°) End-of-Bracing ATR (°) Final Follow-Up ATR (°) Δ ATR (°) (% change)	10 6	17 5	15 10	17 7	14.75 ± 3.3 7.0 ± 2.2
	6	9	8	6	7.25 ± 1.5
	−4 (40)	−8 (47.1)	−7 (46.7)	−11 (64.7)	−7.5 ± 2.9

**Figure A1:**
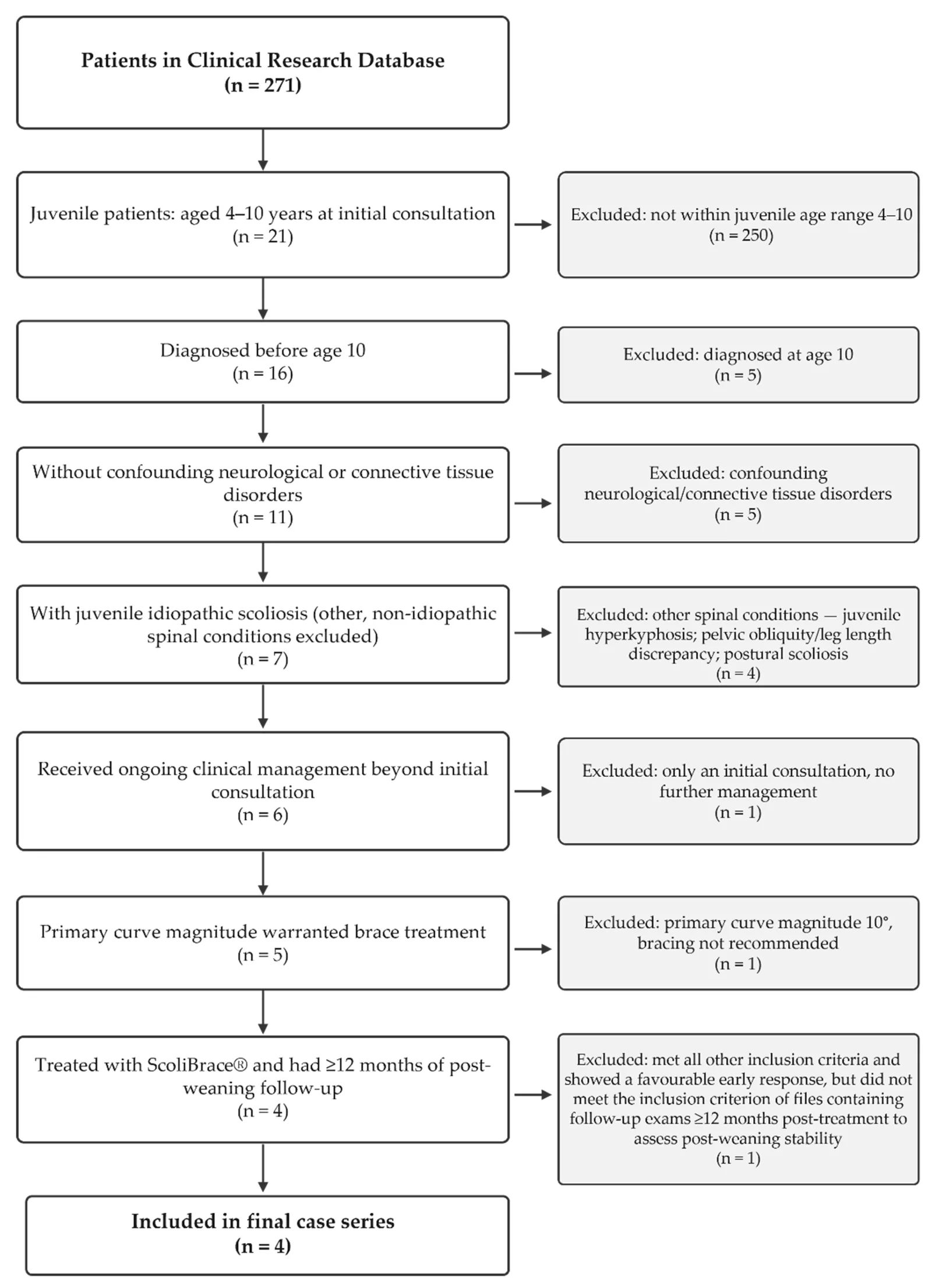
Participant selection flowchart from the clinical research database (*n* = 271) to the final sample included (*n* = 4).

**Figure A2:**
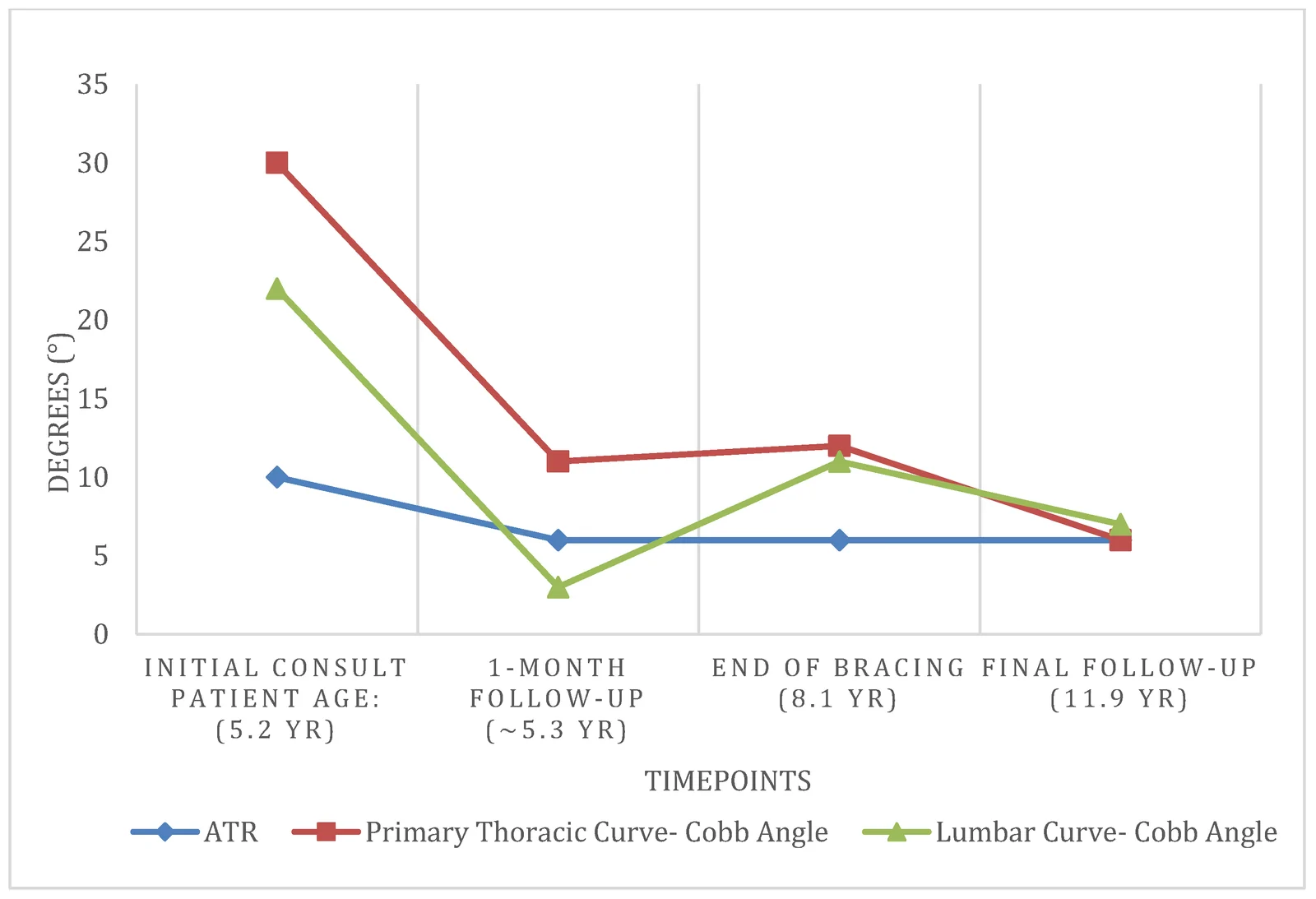
Patient 1: Primary and lumbar Cobb angle and angle of trunk rotation (ATR) measures at key treatment stages: initial consultation, 1-month follow-up, end of bracing, and final follow-up. Patient age (years) at each stage is shown in parentheses on the x-axis. Patient 1 was first diagnosed in November 2017, at approximately 4.7 years of age.

**Figure A3:**
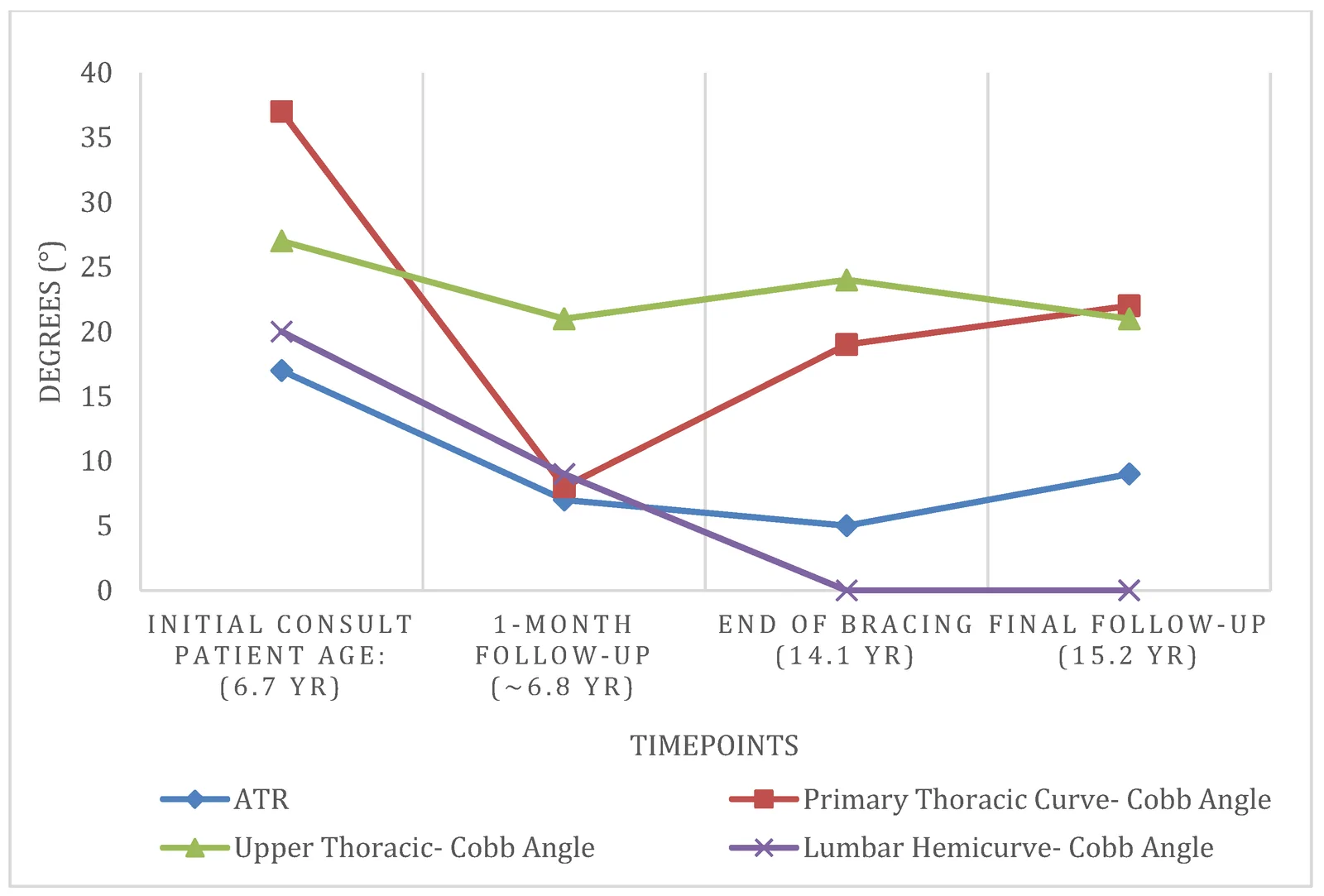
Patient 2: Primary, upper thoracic, and lumbar hemicurve Cobb angle and ATR measures at key treatment stages: initial consultation, 1-month follow-up, end of bracing, and final follow-up. Patient age (years) at each stage is shown in parentheses on the x-axis. Patient 2 was first diagnosed in March 2016, at approximately 5.8 years of age.

**Figure A4:**
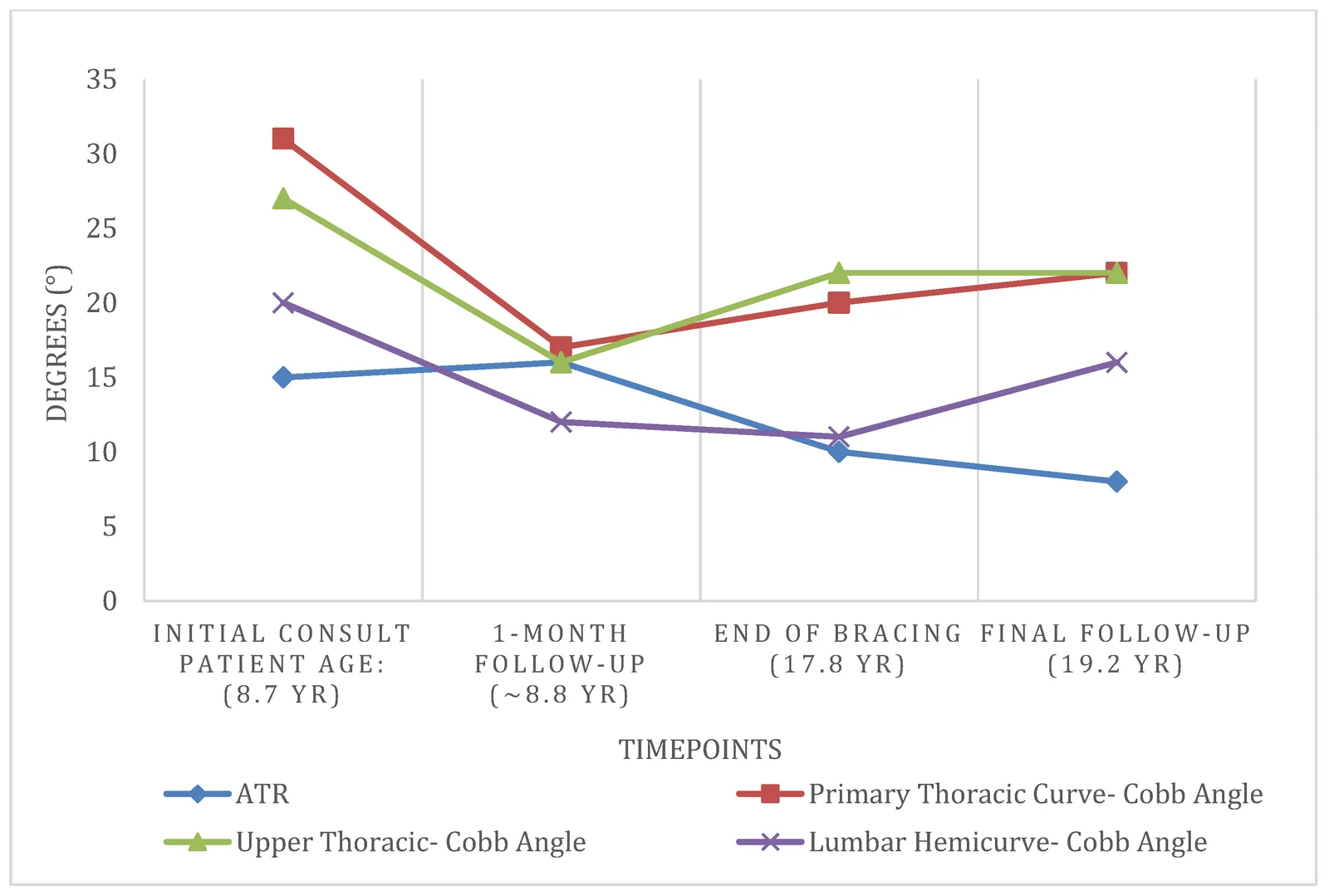
Patient 3: Primary, upper thoracic, and lumbar hemicurve Cobb angle and ATR measures at key treatment stages: initial consultation, 1-month follow-up, end of bracing, and final follow-up. Patient age (years) at each stage is shown in parentheses on the x-axis. Patient 3 was first diagnosed in November 2013, at approximately 8.1 years of age.

**Figure A5:**
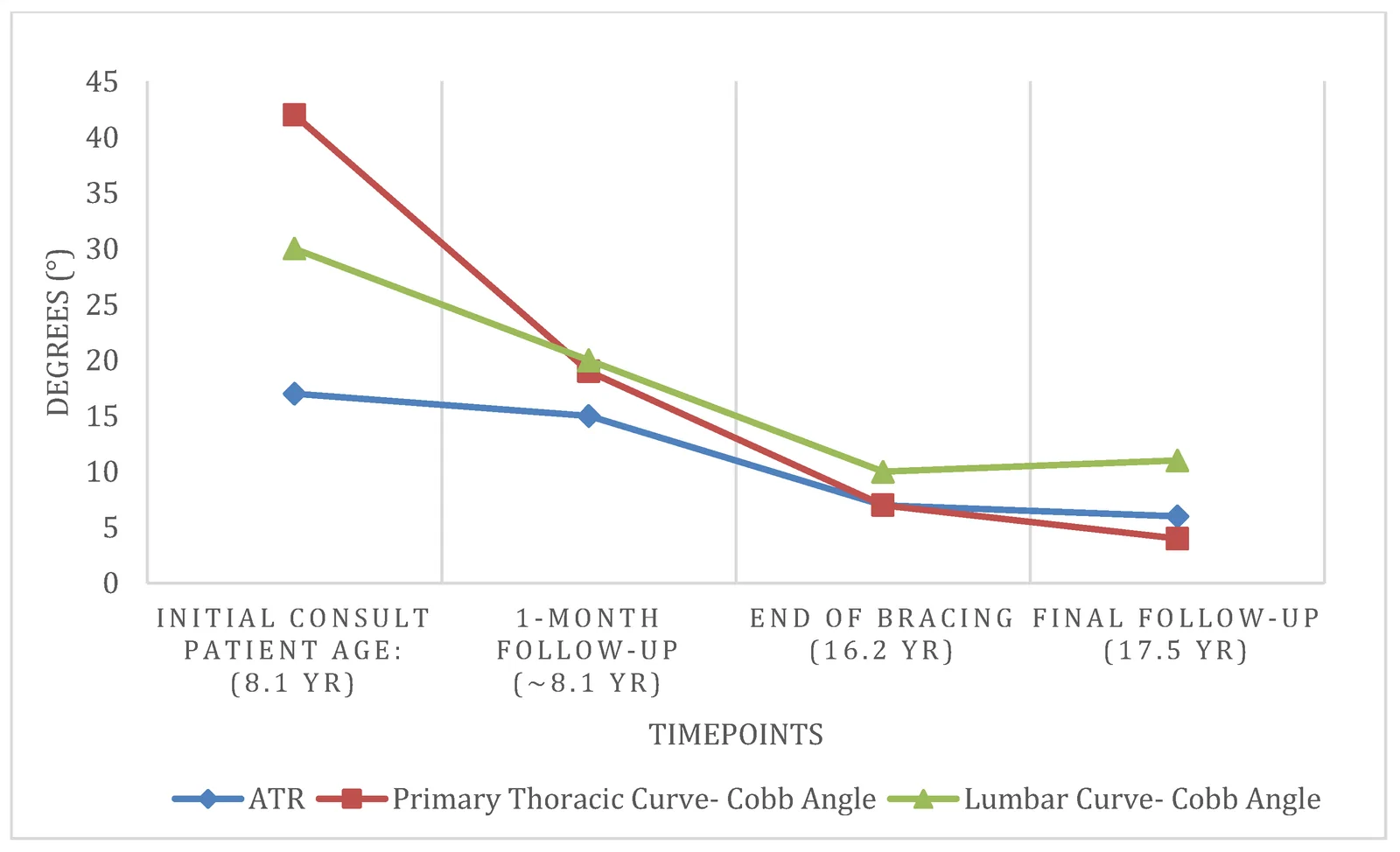
Patient 4: Primary and lumbar Cobb angle and angle of trunk rotation (ATR) measures at key treatment stages: initial consultation, 1-month follow-up, end of bracing, and final follow-up. Patient age (years) at each stage is shown in parentheses on the x-axis. Patient 4 was first diagnosed at 6 years of age; the specific month is unknown.

## Data Availability

The data presented in this study are available from the corresponding author upon reasonable request, subject to privacy and ethical restrictions and the conditions of the approved research ethics protocol.

## Abbreviations

The following abbreviations are used in this manuscript:

AIS	Adolescents with Idiopathic Scoliosis
ATR	Angle of Trunk Rotation
CAD/CAM	Computer-Aided Design and Manufacturing
ICD-10	International Classification of Diseases, Tenth Revision
JIS	Juvenile Idiopathic Scoliosis
L1–L5	Lumbar 1–5
PA	Posteroanterior
PSSE	Physiotherapeutic Scoliosis-Specific Exercise
SD	Standard Deviation
T1–T12	Thoracic 1–12
TLSO	Thoracolumbosacral Orthosis

## Appendix A

### Appendix A.1. Participant Selection

Of the 271 patients in the clinical research database, 21 were aged 4–10 years at initial consultation, the age range used to define JIS. Of these, five were diagnosed at age 10 and were excluded, as the diagnosis was not established prior to this age. A further five patients were excluded due to confounding neurological or connective tissue disorders, and four were excluded for other, non-idiopathic spinal conditions (juvenile hyperkyphosis; pelvic obliquity with leg length discrepancy; postural scoliosis). One patient had only an initial consultation with no further clinical management, and one patient’s primary curve magnitude (10°) did not warrant bracing. One further patient met all other inclusion criteria and demonstrated a favorable early response to bracing (Cobb angle improvement from 22° to 12° at the 1-month brace review), but did not meet the inclusion criterion of files containing follow-up exams ≥12 months post-treatment to assess post-weaning stability, and was therefore excluded. This yielded a final sample of four patients who were treated with ScoliBrace^®^ and had a minimum of 12 months of post-weaning follow-up.

### Appendix A.2. Individual Patient Cobb Angle and ATR Changes Across Treatment Timepoints

This appendix presents individual patient-level Cobb angle and ATR data at key timepoints throughout each patient’s treatment course, depicting the trajectory of curve and rotational changes over time (Figure A2, Figure A3, Figure A4 and Figure A5). For Patients 1 and 4, primary thoracic and lumbar curves are reported; for Patients 2 and 3, primary thoracic, upper thoracic, and a lumbar hemicurve are reported.
